# Individualized Medication Selection and Outcome Prediction for Patients With Dementia and BPSD Based on Systemic Immune-Inflammation Index (SII): A Retrospective Case-Control Study

**DOI:** 10.62641/aep.v54i4.2229

**Published:** 2026-08-15

**Authors:** Jiawen Liu, Haoshui Hua, Qicheng Zhang, Yuming Jiang, Mengting Li

**Affiliations:** ^1^Department of Geriatric Psychiatry, The Third People’s Hospital of Fuyang, 311400 Hangzhou, Zhejiang, China; ^2^Department of Medical Education, The Third People’s Hospital of Fuyang, 311400 Hangzhou, Zhejiang, China

**Keywords:** dementia, antipsychotic agents, biomarkers, inflammation, risperidone

## Abstract

**Background:**

Behavioural and psychological symptoms of dementia (BPSD) are often treated with atypical antipsychotics, but heterogeneous treatment responses remain a clinical challenge. A systemic inflammatory state may contribute to this heterogeneity, but it is unclear whether the baseline systemic immune-inflammation index (SII) is associated with differential short-term responses among BPSD patients treated with risperidone, olanzapine, and quetiapine. Therefore, this study aimed to investigate whether baseline SII was associated with differential short-term responses among antipsychotic treatment groups.

**Methods:**

This was a retrospective cohort study. We reviewed the electronic medical records of hospitalised patients with dementia and BPSD from January 2020 to December 2024 and divided the patients into a risperidone group, an olanzapine group and a quetiapine group according to the antipsychotic treatment in the medical records. Using the risperidone group as the reference, 1:1 nearest-neighbour matching without replacement was performed to obtain three balanced groups of 77 patients each (n = 231 in total). Normally distributed continuous data were analysed using t-tests, analysis of variance (ANOVA) and paired t-tests; non-normally distributed continuous data were analyzed using Wilcoxon rank-sum tests, Kruskal–Wallis tests and Wilcoxon signed-rank tests; categorical data were analysed using chi-square tests. Correlation analysis, multivariate linear regression and restricted cubic splines (RCS) were used for additional analyses.

**Results:**

Spearman’s rank correlation test revealed divergent therapeutic trajectories. Baseline SII was positively correlated with the reduction rate of the Scale for the Assessment of Positive Symptoms (SAPS) in the risperidone group (r = 0.863, *p* < 0.001), and negatively correlated with the reduction rate of the SAPS in the olanzapine group (r = –0.513, *p* < 0.001) and the quetiapine group (r = –0.368, *p* < 0.001). RCS analysis further revealed a non-linear relationship between SII and SAPS reduction rate (non-linear test *p* = 0.001). A significant interaction between medication type and SII was confirmed by the multivariable regression model (interaction *p* < 0.001).

**Conclusions:**

Baseline systemic inflammatory status is differentially associated with short-term antipsychotic response in hospitalised patients with dementia and BPSD. These findings suggest that inflammatory burden may be clinically relevant to the heterogeneous short-term response patterns across antipsychotic groups. However, prospective validation is required before any implications for treatment stratification can be drawn.

## Introduction

The contemporary global health landscape is grappling with the relentless emergence of dementia, including Alzheimer’s disease and related dementias (ADRD). The World Health Organization anticipates that this challenge will affect an astonishing 139 million people by 2050 [1]. In addressing this crisis, it is not sufficient to view it from the perspective of cognitive decline; the high prevalence of behavioural and psychological symptoms of dementia (BPSD) must also be recognised. A recent systematic review and meta-analysis conducted in acute hospital settings reported that approximately 60% of older patients with dementia exhibit at least one BPSD during admission, with aggression/agitation occurring in about 39% of cases [2]. This constitutes a complex clinical reality and is a major cause of severe caregiver burnout and the premature and often inevitable transition of patients to institutional care [3].

A sharp discrepancy exists in the clinical treatment of BPSD: on the one hand, there is the gold standard of non-pharmacological treatment, and on the other hand, there is the urgency of clinical safety issues. The application of atypical antipsychotics (AAPs) in BPSD is often hindered by the challenge of balancing efficacy and risk. Schneider’s observation further confirms this reality: although AAPs are statistically superior to placebo, their side effects and limited therapeutic efficacy lead to a discontinuation rate ranging between 16% and 24% [4, 5]. However, treatment efficacy is not uniform across individuals. Perhaps the most concerning issue remains the substantial heterogeneity in individual responses; moreover, antipsychotic use is associated with an increased risk of multiple adverse outcomes, with higher risks in the early stages after the start of treatment [6].

Although antipsychotic drugs remain widely used in geriatrics worldwide for the treatment of BPSD in dementia, several problems persist, most notably the absence of validated biomarkers that could truly guide and personalise drug selection [7]. The current reliance on heuristic and ad hoc prescribing practices exposes older adults—particularly those with dementia—to a substantial risk of off-target drug effects. Therefore, identifying objective and readily available indicators that capture subtle interindividual differences in treatment responses among individuals is crucial for the future advancement of geriatric psychiatry.

The intricate interplay between the peripheral immune environment and central nervous system homeostasis is attracting increasing attention in immunopsychiatry. Increasing evidence suggests that peripheral inflammatory dysregulation is linked to neurodegenerative vulnerability and cognitive impairment, rather than being a mere epiphenomenon [8, 9]. It is precisely this complexity that has motivated the introduction of the systemic immune-inflammation index (SII)—a biomarker integrating dynamic changes in neutrophils, lymphocytes, and platelets—into this context [10]. Elevated SII values suggest an increased burden of small vessel disease and neuropsychiatric vulnerability [9]; moreover, inflammatory signaling may interact with dopaminergic and microglial pathways involved in behavioural disturbances and treatment response [11]. However, in clinical practice, whether baseline inflammatory features truly correlate with variability in the efficacy of AAPs for BPSD remains unexplored by empirical research.

In this retrospective cohort study, we aimed to investigate whether baseline SII is associated with differences in short-term response and safety profile among elderly patients with BPSD treated with risperidone, olanzapine, or quetiapine. We hypothesized that the baseline inflammatory status is associated with the heterogeneity of therapeutic responses across different drugs.

## Methods

### Study Design and Participants

#### Study Design

Grounded in the ethical framework articulated by the Declaration of Helsinki, this retrospective cohort study used routinely documented inpatient medical records that were stripped of all personal identifiers and consolidated into a fully anonymized dataset under restricted access. The study was reviewed and approved by the Institutional Review Board of The Third People’s Hospital of Fuyang (ethical approval number: (2026)-(2-003-01)), and written informed consent was obtained from all participants according to institutional requirements.

#### Cohort Construction and Matching Strategy

This study is a retrospective analysis based on clinical data routinely recorded in electronic health records. Between January 2020 and December 2024, hospitalised patients with dementia and clinically significant BPSD were retrospectively identified from routinely documented electronic medical records. To create comparable groups while preserving real-world treatment assignment, we employed a reference-group matching design [12]. We first identified eligible patients who received risperidone and selected 77 of them to serve as the fixed reference group. Then, for each of the 77 risperidone patients, we performed two independent 1:1 nearest-neighbour matchings without replacement, identifying one matched patient from both the olanzapine and quetiapine groups. The matching variables included standard demographic factors such as age (within a margin of 2 years), education, and body mass index (BMI), as well as more detailed clinical characteristics including dementia subtype, disease duration, swallowing ability, and a range of comorbidities such as prior stroke and chronic obstructive pulmonary disease.

#### Inclusion and Exclusion Criteria

Inclusion criteria: (1) Aged ≥ 65 years; (2) Diagnosis of Alzheimer’s disease (AD), vascular dementia (VaD), or mixed dementia according to DSM-5 (Diagnostic and Statistical Manual of Mental Disorders, Fifth Edition) or ICD-10 (International Statistical Classification of Diseases and Related Health Problems, 10th Revision) criteria; (3) Significant BPSD present at admission and complete Scale for the Assessment of Positive Symptoms (SAPS) scores available; (4) Monotherapy with risperidone, olanzapine, or quetiapine for at least 14 days.

Exclusion criteria: (1) Missing baseline counts of neutrophil, lymphocyte, or platelet counts; (2) Presence of acute infection, severe hematological disease, or use of immunosuppressants or corticosteroids within the past 3 months; (3) Inability to take oral medications due to severe dysphagia or other physical conditions; (4) Length of stay of less than 14 days.

### Clinical Variables and Data Extraction

Baseline variables were extracted from clinical evaluations and laboratory results recorded within 24 hours of admission. The outcome assessment was based on discharge records following at least 14 days of antipsychotic monotherapy during the index hospitalisation. Therefore, in this study, “short-term” refers to inpatient outcomes during the index hospitalisation rather than to a fixed follow-up period.

Blood test results recorded within 24 hours of admission were also extracted from the medical records. The SII was calculated using the following formula: SII = (Platelet × Neutrophil)/Lymphocyte [10].

Scores from the Scale for the Assessment of Positive Symptoms (SAPS) [13], extracted from medical records, served as a pragmatic measure of psychosis-related symptom burden. SAPS is a clinician-rated tool containing 34 items, with item scores ranging from 0 (none) to 5 (severe), yielding a total score ranging from 0 to 170. Higher total scores indicate more severe psychotic symptoms. This scale has demonstrated good clinical reliability and construct validity for assessing positive psychotic symptoms in various clinical populations. SAPS was originally developed for use in schizophrenia; however, it was used here as a proxy measure for psychosis-dominant BPSD presentations rather than as a dementia-specific, validated BPSD instrument. Based on the outcome definition provided by the Psychiatric Symptom Scale [14], the primary continuous outcome was defined as SAPS reduction rate, calculated according to the following formula: SAPS reduction rate (%) = (SAPS_Admission – SAPS_Discharge) / SAPS_Admission × 100.

Scores from the Mini-Mental State Examination (MMSE) were extracted from medical records to quantitatively assess patients’ cognitive status [15]. The scale covers multiple cognitive domains, including orientation, attention, and memory, yielding a total score ranging from 0 to 30. Lower scores indicate more severe cognitive impairment. This scale is one of the most widely used cognitive screening tools in dementia populations, and its reliability and construct validity have been well established. It is therefore suitable for assessing the cognitive status of the patients in this study [16]. Dementia severity was categorized based on admission MMSE score into three levels: mild (21–25), moderate (15–20), and severe (≤ 14) cognitive impairment. This three-level classification was derived from previously reported MMSE-based severity groupings, in which the categories of moderately severe (10–14) and severe (0–9) dementia were merged into a single severe (≤ 14) category for analytical purposes in the present study [17], although MMSE severity cutoffs are not entirely uniform across the literature. In addition, clinical stabilization was operationally defined for the purpose of chart review as follows: (1) absence of rescue sedative medications (e.g., haloperidol or diazepam) for at least three consecutive days; and (2) nursing documentation indicating stable mood, treatment compliance, and the absence of impulsive or self-harm behaviors. This definition served as a pragmatic chart-based inpatient endpoint and was not based on a separately validated external instrument.

Safety outcomes included changes in fasting blood glucose (FBG) during hospitalisation, new-onset pneumonia, extrapyramidal symptoms (EPS), and falls or other bed-related accidents. All adverse events were identified using structured nursing records and physician documentation.

### Statistical Analysis

No imputation procedure was applied for missing data. The final analyses were therefore conducted using complete-case data. All data were analysed using R (Version 4.3.0) (The R Foundation for Statistical Computing, Vienna, Austria). Normality was assessed using the Shapiro–Wilk test. Normally distributed data were presented as mean ± standard deviation (SD), and comparisons were made using analysis of variance (ANOVA) with Bonferroni post-hoc correction; non-normally distributed data were presented as median (Q_1_, Q_3_), and comparisons were made using the Kruskal–Wallis H test with Dunn’s post-hoc test and Holm adjustment. Spearman rank correlation was used to assess the linear association between SII and SAPS reduction rate. We used the variance inflation factor (VIF) to assess multicollinearity, requiring all VIF values to be below 5 as the threshold. To adjust for potential factors, we fitted a multivariate linear regression model to explore the association between the standardized SII and treatment response. The SII was standardized as Z-scores using the formula: Z = (X – mean)/SD, where X represents the raw value. This transformation was applied to improve the comparability of effect estimates and to facilitate the interpretation of regression coefficients. Subsequently, we employed restricted cubic spline (RCS) analysis to examine the potential nonlinear dose-response relationship between SII and the SAPS reduction rate, thereby overcoming the limitations of a simple linear model assumption.

To further explore whether the association between baseline SII and treatment response varied across different antipsychotic medication groups, we conducted an interaction analysis by fitting a multivariate linear regression model. This model included standardized SII, treatment group, and the multiplicative interaction term between standardized SII and treatment group; the risperidone group was designated as the reference group. The SAPS reduction rate served as the dependent variable. The regression coefficient for standardised SII represents the slope of SII in the risperidone group, while the regression coefficient for the interaction term represents the difference in the SII slope between the given treatment group and the risperidone group.

We also conducted an exploratory analysis to compare the incidence of adverse events across the different treatment groups. Furthermore, we conducted a supplementary analysis in which patients within each treatment group were further stratified into “low-SII” and “high-SII” subgroups based on the cohort-wide median baseline SII value (926.9), which exhibited a skewed distribution; the incidence of adverse events was then compared across these subgroups and treatment groups. Given that multiple comparisons were conducted within the same medication group, we applied the Holm correction to adjust the resulting *p*-values. A two-sided *p*-value < 0.05 was considered statistically significant.

## Results

### Baseline Characteristics and Matching Balance

Using risperidone as the reference group, we performed two independent nearest-neighbour 1:1 matching procedures. This yielded 77 matched pairs for the risperidone-olanzapine comparison and 77 matched pairs for the risperidone-quetiapine comparison. These resulted in 77 matched triplets, totaling 231 patients. As shown in Table 1, there were no statistically significant differences observed among the risperidone, olanzapine, and quetiapine groups with respect to demographic variables, dementia subtype or severity, comorbid conditions, baseline laboratory indices, and psychiatric symptom scores (all *p* > 0.05). In the matched sample, baseline SII was right-skewed and was therefore summarised using the median and interquartile range. The cohort median value was 926.9.

**Table 1.** **Baseline demographic and clinical characteristics of the study population (n = 231)**.

Variables		Total (n = 231)	Risperidone (n = 77)	Olanzapine (n = 77)	Quetiapine (n = 77)	Statistic	*p*
BMI (kg/m^2^), Mean ± SD		22.67 ± 1.56	22.67 ± 1.60	22.72 ± 1.51	22.62 ± 1.60	F = 0.07	0.928
Dementia_duration(years), Mean ± SD		4.36 ± 1.65	4.36 ± 1.64	4.32 ± 1.67	4.41 ± 1.65	F = 0.06	0.940
Age (years), M (Q_1_, Q_3_)		79.00 (74.00, 84.50)	79.00 (75.00, 85.00)	79.00 (74.00, 83.00)	79.00 (74.00, 85.00)	H = 0.49	0.783
SII index, M (Q_1_, Q_3_)		926.90 (518.85, 1447.52)	1013.48 (514.58, 1486.51)	926.90 (518.97, 1413.64)	920.00 (521.84, 1455.04)	H = 0.08	0.961
HbA1c admission (%) M (Q_1_, Q_3_)		5.70 (5.50, 6.80)	5.70 (5.50, 6.80)	5.70 (5.50, 6.80)	5.70 (5.50, 6.50)	H = 0.05	0.977
FBG admission (mmol/L), M (Q_1_, Q_3_)		5.40 (5.30, 7.40)	5.30 (5.30, 7.40)	5.40 (5.30, 7.50)	5.40 (5.30, 7.20)	H = 0.21	0.899
MMSE admission (score), M (Q_1_, Q_3_)		17.00 (12.00, 19.00)	17.00 (12.00, 19.00)	17.00 (12.00, 19.00)	17.00 (12.00, 19.00)	H = 0.04	0.981
SAPS admission (score), M (Q_1_, Q_3_)		35.00 (30.00, 39.00)	34.00 (30.00, 39.00)	35.00 (30.00, 39.00)	35.00 (31.00, 38.00)	H = 0.48	0.788
Gender, n(%)						χ^2^ = 0.03	0.983
	Female	115 (49.78)	38 (49.35)	39 (50.65)	38 (49.35)		
	Male	116 (50.22)	39 (50.65)	38 (49.35)	39 (50.65)		
Dementia type, n(%)						χ^2^ = 0.06	1.000
	AD	167 (72.29)	56 (72.73)	56 (72.73)	55 (71.43)		
	VaD	43 (18.61)	14 (18.18)	14 (18.18)	15 (19.48)		
	Mixed	21 (9.09)	7 (9.09)	7 (9.09)	7 (9.09)		
Dementia severity, n(%)						χ^2^ = 0.14	0.998
	Mild	49 (21.21)	16 (20.78)	17 (22.08)	16 (20.78)		
	Moderate	112 (48.48)	38 (49.35)	36 (46.75)	38 (49.35)		
	Severe	70 (30.30)	23 (29.87)	24 (31.17)	23 (29.87)		
Residence status, n(%)						χ^2^ = 0.16	0.922
	Institution	71 (30.74)	23 (29.87)	25 (32.47)	23 (29.87)		
	Community/Home	160 (69.26)	54 (70.13)	52 (67.53)	54 (70.13)		
History of hypertension, n(%)						χ^2^ = 0.04	0.981
	No	83 (35.93)	28 (36.36)	27 (35.06)	28 (36.36)		
	Yes	148 (64.07)	49 (63.64)	50 (64.94)	49 (63.64)		
History of diabetes, n(%)						χ^2^ = 0.30	0.860
	No	167 (72.29)	56 (72.73)	54 (70.13)	57 (74.03)		
	Yes	64 (27.71)	21 (27.27)	23 (29.87)	20 (25.97)		
History of CHD, n(%)						χ^2^ = 0.16	0.921
	No	161 (69.70)	55 (71.43)	53 (68.83)	53 (68.83)		
	Yes	70 (30.30)	22 (28.57)	24 (31.17)	24 (31.17)		
History of stroke, n(%)						χ^2^ = 0.04	0.978
	No	169 (73.16)	56 (72.73)	57 (74.03)	56 (72.73)		
	Yes	62 (26.84)	21 (27.27)	20 (25.97)	21 (27.27)		
History of COPD, n(%)						χ^2^ = 0.07	0.967
	No	196 (84.85)	66 (85.71)	65 (84.42)	65 (84.42)		
	Yes	35 (15.15)	11 (14.29)	12 (15.58)	12 (15.58)		
Swallowing dysfunction, n(%)						χ^2^ = 0.16	0.922
	No	160 (69.26)	54 (70.13)	52 (67.53)	54 (70.13)		
	Yes	71 (30.74)	23 (29.87)	25 (32.47)	23 (29.87)		
Platelet_admission (× 10^9^/L), Mean ± SD		240.42 ± 30.75	238.74 ± 31.69	241.14 ± 29.51	241.36 ± 31.34	F = 0.17	0.843
Neutrophil admission (× 10^9^/L), M (Q_1_, Q_3_)		5.60 (4.32, 6.80)	5.80 (4.30, 6.80)	5.60 (4.30, 6.60)	5.52 (4.32, 6.82)	H = 0.36	0.836
Lymphocyte admission (× 10^9^/L), M (Q_1_, Q_3_)		1.45 (1.10, 1.92)	1.45 (1.10, 1.90)	1.45 (1.15, 1.95)	1.48 (1.12, 1.92)	H = 0.44	0.802
Monocyte admission (× 10^9^/L), M (Q_1_, Q_3_)		0.44 (0.40, 0.47)	0.44 (0.40, 0.48)	0.44 (0.40, 0.47)	0.43 (0.40, 0.47)	H = 0.08	0.963
MLR index, M (Q_1_, Q_3_)		0.27 (0.22, 0.43)	0.27 (0.22, 0.43)	0.27 (0.22, 0.43)	0.27 (0.22, 0.42)	H = 0.38	0.827
Albumin admission (g/L), M (Q_1_, Q_3_)		38.00 (34.50, 40.00)	38.00 (34.20, 40.00)	38.10 (34.50, 40.10)	38.00 (34.00, 40.00)	H = 0.80	0.670
Serum creatinine admission (μmol/L), M (Q_1_, Q_3_)		78.00 (74.00, 92.30)	78.00 (74.00, 92.00)	78.20 (74.20, 92.10)	78.00 (74.00, 92.50)	H = 0.36	0.836
Education years, M (Q_1_, Q_3_)		9.00 (6.00, 12.00)	9.00 (6.00, 12.00)	9.00 (6.00, 12.00)	9.00 (6.00, 12.00)	H = 0.92	0.631

Note: F, Analysis of Variance (ANOVA); H, Kruskal–Wallis test; χ^2^, chi-square test; SD, standard deviation; M, median; Q_1_, 1st Quartile; Q_3_, 3rd Quartile. Abbreviations: BMI, Body Mass Index; SII, Systemic Immune-Inflammation Index; MLR, Monocyte-to-Lymphocyte Ratio; FBG, Fasting Blood Glucose; HbA1c, Glycated Hemoglobin; SAPS, Scale for the Assessment of Positive Symptoms; MMSE, Mini-Mental State Examination; COPD, Chronic Obstructive Pulmonary Disease; CHD, Coronary Heart Disease. Dementia severity was categorized according to admission MMSE score as mild (21–25), moderate (15–20), or severe (≤14) cognitive impairment.

### Changes in Clinical Symptoms

Within-group analysis showed that after treatment with risperidone, olanzapine, or quetiapine, SAPS scores and FBG levels significantly changed compared with admission (all *p* < 0.001). MMSE scores showed no significant between-group differences at admission or at discharge. In the within-group paired analyses, a small but statistically significant change in MMSE scores was observed only in the olanzapine group (Table 2). For between-group comparisons of clinical change, change scores (discharge minus admission) were analysed (Table 3). The Kruskal–Wallis test showed a significant difference in SAPS change scores among the three groups (*p* < 0.001). Dunn’s post-hoc pairwise comparisons showed that the reduction in SAPS scores was greater in the risperidone group than in the olanzapine group (*p* = 0.004) and in the quetiapine group (*p* < 0.001). In this study, SAPS-based analyses should be interpreted as reflecting psychosis-related symptom change rather than changes in the full spectrum of BPSD.

**Table 2.** **Within-group changes in psychiatric symptoms, cognitive function, and fasting blood glucose from admission to discharge**.

Variables	Group	Admission	Discharge	Wilcoxon signed-rank test statistic	*p*
	Risperidone	5.30 (5.30, 7.40)	5.80 (5.50, 7.10)	239.00	<0.001
FBG	Olanzapine	5.40 (5.30, 7.50)	6.30 (5.80, 7.20)	136.00	<0.001
	Quetiapine	5.40 (5.30, 7.20)	5.80 (5.50, 6.80)	258.50	<0.001
	H	0.21	9.01		
	*p*	0.899	0.011		
	Risperidone	17.00 (12.00, 19.00)	17.00 (12.00, 19.00)	563.00	0.272
MMSE	Olanzapine	17.00 (12.00, 19.00)	17.00 (12.00, 18.00)	680.50	0.004
	Quetiapine	17.00 (12.00, 19.00)	17.00 (15.00, 19.00)	363.00	0.262
	H	0.04	1.09		
	*p*	0.981	0.579		
	Risperidone	34.00 (30.00, 39.00)	19.00 (17.00, 21.00)	3003.00	<0.001
SAPS	Olanzapine	35.00 (30.00, 39.00)	22.00 (21.00, 24.00)	3003.00	<0.001
	Quetiapine	35.00 (31.00, 38.00)	27.00 (26.00, 29.00)	2704.00	<0.001
	H	0.48	171.84		
	*p*	0.788	<0.001		

Note: H, Kruskal–Wallis test. Abbreviations: SAPS, Scale for the Assessment of Positive Symptoms; FBG, fasting blood glucose; MMSE, Mini-Mental State Examination.

**Table 3.** **Post-hoc pairwise comparisons of discharge-minus-admission clinical change scores among treatment groups**.

Variables	Total (n = 231)	Risperidone (n = 77)	Olanzapine (n = 77)	Quetiapine (n = 77)	Kruskal–Wallis test	*p*	Post-hoc Dunn Test (*p* adjusted)
FBG fluctuation, (Dis-Adm)	0.50 (0.20, 0.75)	0.50 (0.20, 0.70)	0.70 (0.20, 1.10)	0.40 (0.20, 0.70)	12.90	0.002	Risp vs Ola (0.008); Risp vs Que (0.829); Ola vs Que (0.004)
MMSE change, (Dis-Adm)	0.00 (–1.00, 0.00)	0.00 (–1.00, 0.00)	0.00 (–1.00, 0.00)	0.00 (–1.00, 1.00)	6.36	0.042	Risp vs Ola (0.533); Risp vs Que (0.723); Ola vs Que (0.035)
SAPS change, (Dis-Adm)	–11.00 (–16.00, –7.00)	–16.00 (–20.00, –11.00)	–11.00 (–17.00, –8.00)	–7.00 (–11.00, –3.00)	67.18	<0.001	Risp vs Ola (0.004); Risp vs Que (<0.001); Ola vs Que (<0.001)

Note: Abbreviations: SAPS, Scale for the Assessment of Positive Symptoms; FBG, fasting blood glucose; MMSE, Mini-Mental State Examination; Adm, admission; Dis, discharge; Risp, risperidone; Ola, olanzapine; Que, quetiapine; vs, versus. For post-hoc multiple comparisons, Dunn’s test with Holm adjustment was used for nonparametric pairwise comparisons. Negative SAPS change values indicate symptom improvement.

Regarding cognitive function and glucose metabolism, there were statistically significant differences in MMSE change scores among the three groups (*p* = 0.042). However, Dunn’s post-hoc analysis showed a statistically significant but numerically small difference between the olanzapine and quetiapine groups (*p* = 0.035), with no significant differences observed for the other comparisons. There were significant differences in FBG change scores among the three groups (*p* = 0.002). Dunn’s post-hoc comparison showed that the increase in blood glucose levels in the olanzapine group was greater than those in the risperidone group (*p* = 0.008) and the quetiapine group (*p* = 0.004).

### Association Between Baseline SII and Treatment Response

**Fig. 1.**
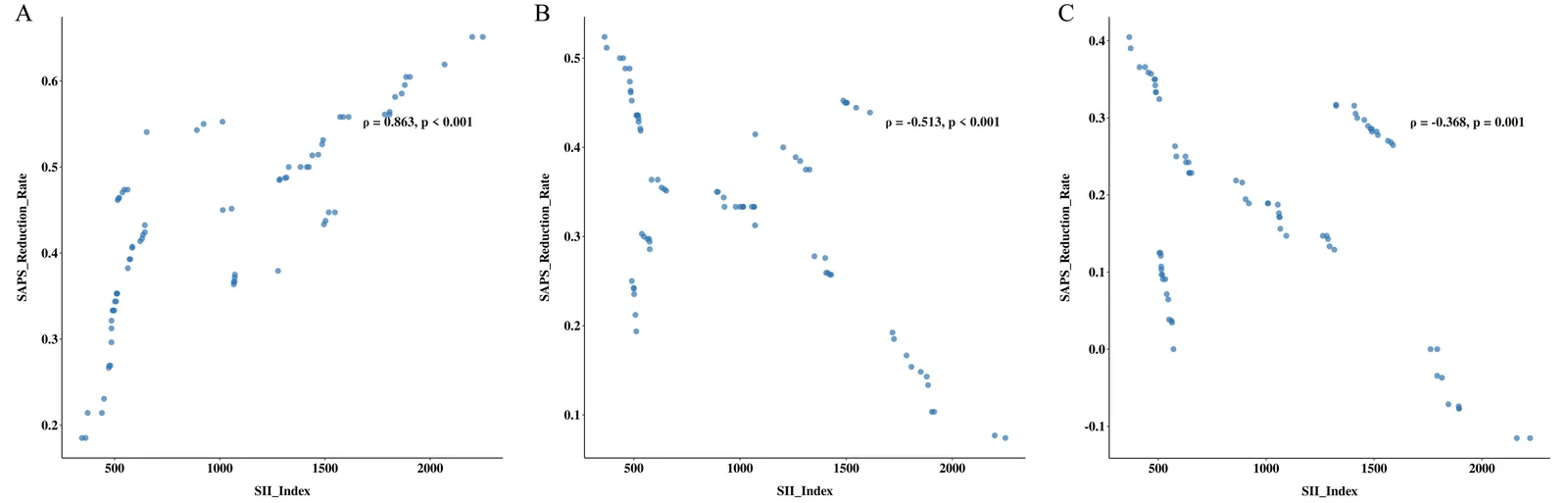
**Correlation analysis between baseline SII and SAPS reduction rate**. (A: Risperidone, Spearman’s correlation coefficient r = 0.863, *p* < 0.001; B: Olanzapine, Spearman’s correlation coefficient r = –0.513, *p* < 0.001; C: Quetiapine, Spearman’s correlation coefficient r = –0.368, *p* = 0.001. These analyses describe the direction of associations within treatment groups and are presented for exploratory purposes.)

Fig. 1 illustrates the positive correlation between the rate of SAPS score reduction and baseline SII in the risperidone group (r = 0.863, *p* < 0.001). Olanzapine and quetiapine both showed negative correlations of different magnitudes (r = –0.513 and r = –0.368, both *p* < 0.001).

### Multivariable Regression and Dose–response Analysis

**Fig. 2.**
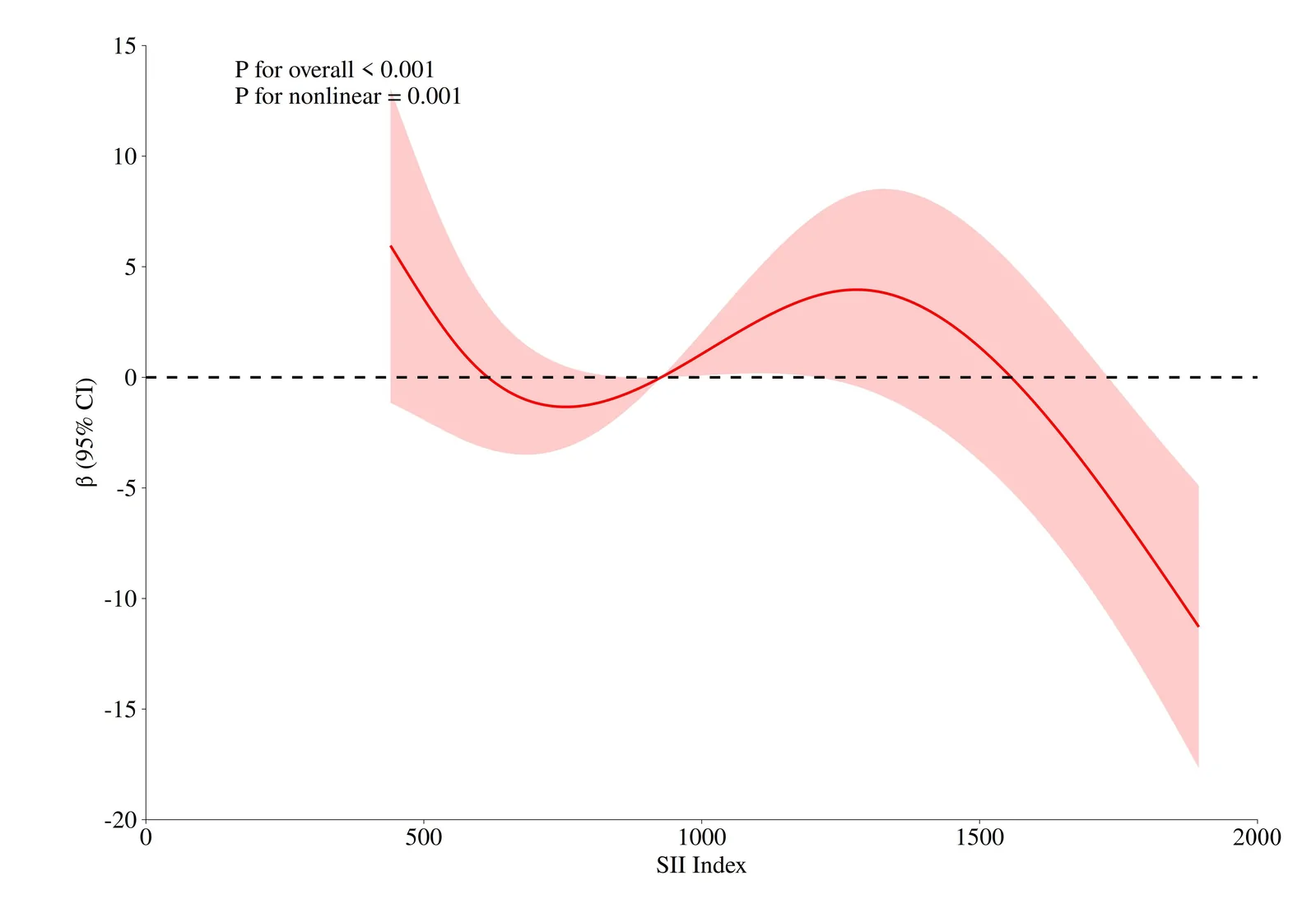
**Dose-response relationship between SII and therapeutic efficacy using Restricted Cubic Spline (RCS) regression**.

In the multivariable linear regression model, standardised SII remained significantly associated with SAPS reduction rate after adjustment for demographic and clinical covariates (β = –4.60, 95% CI: –7.45 to –1.75, *p* = 0.002; Table 4). Additionally, RCS analysis revealed a clearly nonlinear relationship between baseline SII and symptomatic change (*p* for nonlinearity = 0.001; Fig. 2).

**Table 4.** **Multivariable linear regression analysis of the association between standardised SII and SAPS reduction rate**.

Variables		S.E	t	*p*	β (95%CI)
SII index stand group		1.45	–3.17	0.002	–4.60 (–7.45 to –1.75)
Risperidone					0.00 (Reference)
Olanzapine		1.94	–5.38	<0.001	–10.44 (–14.24 to –6.64)
Quetiapine		1.92	–13.31	<0.001	–25.60 (–29.37 to –21.83)
Gender					
	Female				0.00 (Reference)
	Male	1.64	1.26	0.209	2.06 (–1.15 to 5.26)
Dementia type					
	AD				0.00 (Reference)
	VaD	2.37	–0.47	0.640	–1.11 (–5.76 to 3.54)
	Mixed	3.63	0.88	0.379	3.20 (–3.91 to 10.32)
Residence status					
Institution					0.00 (Reference)
Community/Home		4.10	–0.94	0.351	–3.84 (–11.88 to 4.20)
History of hypertension					
	No				0.00 (Reference)
	Yes	3.21	–0.84	0.400	–2.71 (–8.99 to 3.58)
History of diabetes					
	No				0.00 (Reference)
	Yes	2.22	0.72	0.471	1.61 (–2.75 to 5.96)
History of CHD					
	No				0.00 (Reference)
	Yes	2.23	–1.9	0.059	–4.24 (–8.61 to 0.13)
History of stroke					
	No				0.00 (Reference)
	Yes	3.86	0.08	0.933	0.32 (–7.24 to 7.89)
History of COPD					
	No				0.00 (Reference)
	Yes	3.74	–1.37	0.171	–5.14 (–12.47 to 2.19)
Swallowing dysfunction					
	No				0.00 (Reference)
	Yes	2.51	–1.21	0.228	–3.04 (–7.96 to 1.88)
Age		0.27	–0.13	0.893	–0.04 (–0.57 to 0.50)
BMI		1.08	–1.63	0.105	–1.76 (–3.87 to 0.36)
Dementia duration		1.12	–0.17	0.868	–0.19 (–2.38 to 2.01)
MMSE admission		0.69	–1.34	0.183	–0.92 (–2.27 to 0.43)

AD, Alzheimer’s disease; VaD, vascular dementia; CHD, Coronary Heart Disease; COPD, Chronic Obstructive Pulmonary Disease; BMI, Body Mass Index; MMSE, Mini-Mental State Examination.

The model describes the association between the SII and SAPS reduction rate, adjusted for all covariates listed in Table 4. The solid line represents the estimated fitted values, and the shaded region represents the 95% confidence interval. Knots were placed at the 10th, 50th, and 90th percentiles based on the Bayesian Information Criterion (BIC).

### Interaction and Subgroup Analyses

In the multivariable interaction model with risperidone as the reference group, standardised SII was positively associated with SAPS reduction rate in the risperidone group (β = 0.05, 95% CI: 0.02 to 0.08, *p* < 0.001). Compared to the risperidone group, the SII slope was significantly lower in the olanzapine group (interaction β = –0.16, 95% CI: –0.19 to –0.13, *p* < 0.001) and in the quetiapine group (interaction β = –0.14, 95% CI: –0.17 to –0.11, *p* < 0.001). The overall interaction test confirmed a significant effect modification by treatment group (*p* for interaction < 0.001) (Table 5).

**Table 5.** **Multivariable interaction analysis of baseline standardised SII and treatment group for SAPS reduction rate**.

Variables	β	95% CI	*p*
Standardized SII (slope in risperidone group)	0.05	0.02 to 0.08	<0.001
Standardized SII × Olanzapine (vs risperidone)	–0.16	–0.19 to –0.13	<0.001
Standardized SII × Quetiapine (vs risperidone)	–0.14	–0.17 to –0.11	<0.001
Overall *p* for interaction	—	—	<0.001

Note: The outcome variable was SAPS reduction rate. SII was Z-score standardised. Risperidone was used as the reference group. The coefficient for standardised SII represents the slope of SII in the risperidone group, whereas the interaction terms represent the difference in the SII slope between the given treatment group and the risperidone group. The model was adjusted for age, sex, dementia type, residential status, history of hypertension, diabetes, coronary heart disease, stroke, chronic obstructive pulmonary disease, swallowing dysfunction, BMI, dementia duration, and admission MMSE score.

Among patients in the high-SII subgroup, the SAPS reduction rate differed across treatment groups, with the risperidone group showing the highest reduction rate (Table 6). In patients with low baseline SII, the difference in SAPS reduction rates between treatment groups was smaller. The number of days required to achieve clinical stabilisation also varied among treatment groups. In the high-SII subgroup, patients in the risperidone group reached clinical stabilisation earlier than those in the olanzapine or quetiapine groups.

**Table 6.** **Subgroup analysis of therapeutic efficacy and safety outcomes stratified by baseline SII levels**.

Subgroup	Variables	Total	Risperidone	Olanzapine	Quetiapine	Statistic	*p*
Low-SII	SAPS_reduction_rate, Mean ± SD	0.32 ± 0.13	0.37 ± 0.10	0.38 ± 0.09	0.21 ± 0.12	F = 30.93	<0.001
	FBG fluctuation, M (Q_1_, Q_3_)	0.20 (–0.10, 0.30)	0.20 (–0.18, 0.30)	0.25 (0.20,1.32)	0.20 (–0.15, 0.30)	χ^2^ = 9.83^#^	0.007
	Days to stabilization, M (Q_1_, Q_3_)	6.00 (6.00, 7.00)	5.00 (5.00, 6.00)	7.00 (6.00, 7.00)	7.00 (6.00, 7.00)	χ^2^ = 45.02^#^	<0.001
High-SII	SAPS_reduction_rate, Mean ± SD	0.32 ± 0.19	0.51 ± 0.08	0.29 ± 0.12	0.15 ± 0.14	F = 89.70	<0.001
	FBG fluctuation, M (Q_1_, Q_3_)	0.70 (0.60, 1.10)	0.70 (0.60,0.85)	0.70 (0.60, 1.10)	0.70 (0.50, 0.88)	χ^2^ = 2.62^#^	0.270
	Days to stabilization, M (Q_1_, Q_3_)	7.00 (4.00, 8.00)	4.00 (4.00,4.00)	8.00 (7.00, 8.00)	8.00 (7.00, 9.00)	χ^2^ = 62.10^#^	<0.001

Note: Patients were stratified into Low SII (< 926.9) and High SII (≥ 926.9) subgroups. Abbreviations: FBG, Fasting Blood Glucose; SAPS, Scale for the Assessment of Positive Symptoms. F, ANOVA; ^#^, Kruskal–Wallis test; SD, standard deviation; M, median; Q_1_, 1st Quartile; Q_3_, 3rd Quartile.

### Safety Outcomes

There were no significant differences in the incidence of new-onset pneumonia or falls among the three treatment groups. However, the incidence of extrapyramidal symptoms was higher in the risperidone group (19.48%) compared to the olanzapine and quetiapine groups (1.30% each) (*p* < 0.001) ((Table 7)).

**Table 7.** **Comparison of safety profiles and adverse events among the three treatment groups**.

Variables		Total (n = 231)	Risperidone (n = 77)	Olanzapine (n = 77)	Quetiapine (n = 77)	Statistic	*p*
New onset pneumonia, n (%)						χ^2^ = 0.16	0.924
	No	183 (79.22)	61 (79.22)	62 (80.52)	60 (77.92)		
	Yes	48 (20.78)	16 (20.78)	15 (19.48)	17 (22.08)		
EPS, n (%)						χ^2^ = 24.89	<0.001
	No	214 (92.64)	62 (80.52)	76 (98.70)	76 (98.70)		
	Yes	17 (7.36)	15 (19.48)	1 (1.30)	1 (1.30)		
Falls and bed drops, n (%)						χ^2^ = 0.70	0.704
	No	209 (90.48)	71 (92.21)	68 (88.31)	70 (90.91)		
	Yes	22 (9.52)	6 (7.79)	9 (11.69)	7 (9.09)		

Note: χ^2^, chi-square test. Falls and bed drops included any recorded incidents during the hospitalisation period. Abbreviations: EPS, Extrapyramidal Symptoms (including tremor, rigidity, and akathisia).

Further safety analyses stratified by baseline SII within each treatment group showed that, after Holm adjustment for multiple comparisons, patients with high baseline SII had consistently higher rates of new-onset pneumonia across all three antipsychotic groups. The excess risk of extrapyramidal symptoms associated with high SII was mainly observed in the risperidone group, whereas no significant subgroup difference was observed in the olanzapine or quetiapine groups. No significant SII-related difference was observed for falls or other bed-related accidents within any treatment group (**Supplementary Table 1**).

## Discussion

In this retrospective cohort study of elderly patients with BPSD, we investigated whether baseline systemic inflammatory status was associated with differences in short-term response among commonly used atypical antipsychotics. Our findings suggest that baseline SII was differentially associated with short-term symptom change among the three treatment groups. In exploratory subgroup analyses, patients with higher inflammatory burden showed a more favorable short-term symptomatic response pattern in the risperidone group, whereas differences between drugs appeared smaller in patients with lower SII levels. These findings suggest heterogeneity in treatment-associated response patterns rather than providing definitive drug-selection guidance.

The observed interactions between SII and drug classes in this study may be interpreted hypothetically from the perspective of immune-dopamine interactions. Systemic inflammation increases blood-brain barrier permeability and activates central microglia, thereby altering monoaminergic neurotransmission (including dopaminergic signaling) [18, 19]. In such a hyperinflammatory state, pro-inflammatory cytokines may alter dopamine synthesis, release, and availability [18, 20]. Second, from a pharmacological perspective, risperidone exhibits relatively higher D2 receptor affinity and occupancy, while quetiapine is characterised by rapid dissociation and lower D2 receptor binding affinity [21, 22]. In the presence of stronger inflammation and dopaminergic activity, these differences may be more pronounced, resulting in a more robust response to risperidone in patients with elevated SII. In patients with lower baseline inflammation, this mechanism and pharmacological difference may be masked. However, these biological interpretations were not directly examined in the present study and should be considered hypothesis-generating.

Although risperidone was associated with a more favourable short-term symptom pattern in patients with elevated SII, this favourable pattern was accompanied by a higher rate of extrapyramidal symptoms. In addition, new-onset pneumonia was common in the overall cohort, and, according to exploratory stratified analyses, was more frequent in patients with elevated baseline SII across all three treatment groups. These findings highlight the importance of balancing efficacy with safety, especially for patients with pre-existing swallowing dysfunction. This is because antipsychotic-related dysphagia, extrapyramidal effects and sedation can increase the risk of choking and aspiration pneumonia [23, 24]. Another notable finding was that the SAPS and FBG changed significantly during hospitalisation, whereas the MMSE showed little overall short-term change. This pattern suggests that short-term antipsychotic treatment may alleviate acute psychosis-related behavioural symptoms without conferring a measurable short-term cognitive benefit.

Taken together, our findings suggest that baseline inflammatory burden may be clinically relevant to the heterogeneous response and safety patterns observed across antipsychotic groups. Rather than serving as a standalone predictor or a prescribing tool, SII may help identify patient subgroups suitable for further prospective evaluation. Based on this premise, we propose the following two clinical recommendations: (1) Stratified screening: incorporate SII calculation into routine admission assessments. For patients with elevated SII levels who do not present with significant dysphagia, risperidone may be the preferred agent for achieving rapid symptom control; and (2) De-escalation strategy: for patients with lower SII levels, the selection of quetiapine or olanzapine—agents with superior metabolic and safety profiles—may yield greater clinical benefit.

This study has several limitations. Firstly, despite the use of reference-group matching and multivariable adjustment, the retrospective and non-randomised design implies that residual confounding factors cannot be ruled out, meaning that it is not possible to draw conclusions about cause and effect. Unmeasured factors such as antipsychotic dosage, non-pharmacological interventions, caregiver burden, and baseline functional status may have influenced both efficacy and safety outcomes. Secondly, SAPS was used as a pragmatic proxy for psychosis-related symptoms rather than as a dementia-specific, validated BPSD instrument, and it should not be directly extrapolated to the full spectrum of BPSD. Similarly, “clinical stabilisation” was defined as a practical inpatient outcome derived from medical records rather than independently validated, standardised external instruments. This limitation may reduce the generalisability of the findings. Thirdly, only baseline SII was analysed, and the median SII cutoff point used for subgroup stratification was exploratory in nature rather than clinically validated. Fourthly, the safety stratification analyses were exploratory and based on relatively low event counts. Finally, as this was a single-centre study, the generalisability of its findings is limited. Prospective studies with 6–12 months of follow-up are needed in the future to determine whether inflammation-related subgroup differences are associated with sustained symptom control, relapse risk, and longer-term safety outcomes.

## Conclusions

This study identified a significant interaction between baseline systemic inflammatory status and short-term antipsychotic response patterns in hospitalized patients with BPSD. Patients with an elevated SII may benefit more from risperidone treatment, although this should be balanced against a higher incidence of complications. These findings suggest that SII could serve as a readily available stratification indicator. However, the optimal stratification threshold remains to be determined, and large-scale, multicentre, prospective validation is required before SII can be used to inform prescription decisions.

## Availability of Data and Materials

The data that support the findings of this study are available from the corresponding author upon reasonable request.

## Supplementary Material

Supplementary material associated with this article can be found, in the online version, at https://doi.org/10.62641/aep.v54i4.2229
