# Supplementary material for: Individualized Medication Selection and Outcome Prediction for Patients With Dementia and BPSD Based on Systemic Immune-Inflammation Index (SII): A Retrospective Case-Control Study
Source: Actas Esp Psiquiatr. 2026 Aug 15;54(4):992–1004. doi: 10.62641/aep.v54i4.2229 (PMC13571188; doi:10.62641/aep.v54i4.2229)
Supplement: Supplementary file 1 [file ActEsp-54-4-992-1004-s1.zip › ActEsp-54-4-992-1004.docx]

Supplementary Table S1. Safety outcomes stratified by baseline SII subgroup within each treatment group

| Variables | Low SII risperidone (n=38) | High SII risperidone (n=39) | Adjusted P value | Low SII olanzapine (n=38) | High SII olanzapine (n=39) | Adjusted P value | Low SII quetiapine (n=39) | High SII quetiapine (n=38) | Adjusted P value |
| --- | --- | --- | --- | --- | --- | --- | --- | --- | --- |
| New-onset pneumonia, n (%) | 0 (0.00) | 16 (41.03) | <0.001 | 0 (0.00) | 15 (38.46) | <0.001 | 0 (0.00) | 17 (44.74) | <0.001 |
| Extrapyramidal symptoms, n (%) | 0 (0.00) | 15 (38.46) | <0.001 | 0 (0.00) | 1 (2.56) | 1.000 | 0 (0.00) | 1 (2.63) | 1.000 |
| Falls/bed-related accidents, n (%) | 3 (7.89) | 3 (7.69) | 1.000 | 6 (15.79) | 3 (7.69) | 1.000 | 4 (10.26) | 3 (7.89) | 1.000 |

**Note:** High and low SII subgroups were defined according to the cohort median baseline SII value of 926.9. Categorical variables are presented as n (%) and were compared using Fisher’s exact test. Because multiple within-drug comparisons were performed, P values were adjusted using the Holm method. These analyses were exploratory.
